# miR-182 suppresses invadopodia formation and metastasis in non-small cell lung cancer by targeting cortactin gene

**DOI:** 10.1186/s13046-018-0824-1

**Published:** 2018-07-09

**Authors:** Yongwen Li, Hongbing Zhang, Hao Gong, Yin Yuan, Ying Li, Cong Wang, Weiting Li, Zihe Zhang, Minghui Liu, Hongyu Liu, Jun Chen

**Affiliations:** 1 Department of Lung Cancer Surgery, Tianjin Medical University General Hospital, Anshan Road No.154, Heping District, Tianjin, 300052 China; 2 Tianjin key laboratory of lung cancer metastasis and tumor microenvironment, Tianjin Lung Cancer Institute, Tianjin Medical University General Hospital, Anshan Road No.154, Heping District, Tianjin, 300052 China

**Keywords:** Lung cancer, miRNA-182, Cortactin, Metastasis, Invadopodia

## Abstract

**Background:**

Metastasis is the leading cause of cancer mortality and is a major hurdle for lung cancer treatment. Invadopodia, which are cancer-specific protrusive structures, play a crucial role in the metastatic cascade through degradation of the basement membrane and surrounding stroma. Cortactin, a critical component of invadopodia, frequently used as an invadopodia marker, a universally important player in invadopodia function, and is frequently overexpressed in cancer, but the exact mechanism of regulation is not yet fully understood.

**Methods:**

The expression level of CTTN in human non-small cell lung cancer (NSCLC) tissues was detected by qRT-PCR. Cell migration, invasion and invadopodia formation were assessed in vitro by wound-healing, transwell assay and immunofluorescence, respectively. The dual-luciferase reporter assay was used to identify the direct target of miR-182.

**Results:**

Hepatocyte growth factor (HGF) and phorbol 12,13-dibutyrate (PDBu) can induce CTTN expression, motility, and invasion ability, as well as invadopodia formation in non-small cell lung cancer (NSCLC). Moreover, miR-182 suppressed metastasis and invadopodia formation by targeting CTTN in NSCLC. Our qRT-PCR results showed that CTTN expression was inversely correlated with miR-182 expression that suppressed invadopodia formation via suppression of the Cdc42/N-WASP pathway. Furthermore, miR-182 negatively regulated invadopodia function, and suppressed extracellular matrix(ECM) degradation in lung cancer cells by inhibiting cortactin.

**Conclusion:**

Collectively, our results demonstrated that miR-182 targeted CTTN gene in NSCLC and suppressed lung cancer invadopodia formation, and thus suppressed lung cancer metastasis. This suggests a therapeutic application of miR-182 in NSCLC.

**Electronic supplementary material:**

The online version of this article (10.1186/s13046-018-0824-1) contains supplementary material, which is available to authorized users.

## Background

As the most common cause of cancer-related death worldwide, lung cancer has been a growing problem in China since 2000 due to risk factors such as smoking, air pollution and an aging population [1, 2]. Despite the development of many treatment strategies, the long-term survival rate of lung cancer patients is still very low. The cause of death for the vast majority of cancer patients is the development of metastatic lesions at sites distant from that of the primary tumor. Metastasis is the leading cause of cancer mortality and is a major hurdle for lung cancer treatment.

Metastasis occurs when tumor cells invade basement membranes and blood vessels to colonize other tissues. It is generally agreed that the process of tumor metastasis is a multi-step process and under precise regulation. However, the exact molecular mechanism of metastasis is not fully understood and the molecular pathways underlying each step are still obscure. Invasion of cells through layers of extracellular matrix (ECM) is a key step in tumor metastasis, facilitated by invadopodia, which actin-rich protrusions of the plasma membrane that are associated with the degradation of the ECM in cancer invasiveness and metastasis [3]. By providing direct evidence of the functional importance of invadopodia in cancer cell extravasation, many studies have demonstrated that invadopodia play a crucial role in the metastatic cascade and represent a potential therapeutic target for anti-metastasis strategies [4, 5]. Invadopodia adhesion sites in tumor cells are recognized by dot-like aggregates of actin and cortactin, and their membranes penetrate the matrix in the form of filopodia-like extensions assisted by membrane-associated proteolytic enzymes. In general, invadopodia components fall into two classes of molecules: (1) proteins involved with actin polymerization and membrane remodeling and (2) ECM-degrading proteases. Emerging evidence has revealed a critical role for cortactin in invadopodia as well as in promoting cell motility and invasion [6–8]. Cortactin, plays an important role in actin assembly, scaffolding or cytoskeletal arrangement and membrane trafficking; Cortactin is also a universally important player in invadopodia function, and is likely to be a critical player in invadopodia-associated ECM degradation. As a result, cortactin is frequently used as an invadopodia marker. In addition, several studies have reported that cortactin is often overexpressed in tumors and is associated with metastasis and poor prognosis of patients [9–11]. Cortactin is a potential molecular driver in several cancers, including lung, brain, and colorectal cancer [12, 13].

miRNAs are endogenous and small non-coding RNAs of 20–25 nucleotides in length. They can regulate cell survival, proliferation, differentiation, migration, invasion and metastasis via binding to the 3′ untranslated region (UTR) of some target genes [14]. It has been reported that approximately one-third of human genes may be regulated by miRNAs [15]. Increasing evidence has indicated that miRNAs may function as either oncogenes or tumor suppressors in the malignant progression of various cancers, including lung cancer [14, 16, 17]. As one member of the miR-183/− 96/− 182 cluster, miR-182 has been shown to be directly involved in human cancer processes, such as tumorigenesis, migration and metastasis and to be an important player in regulating tumor progression in various tumors, including lung, brain, and breast tumors [18–22]. However, the roles of miR-182 in different kind of tumors are varied and sometimes contradictory. Therefore, miR-182 may play different roles in diverse kinds of tumor cells.

In this report, we showed that miR-182 inhibited invadopodia formation in NSCLC by targeting CTTN and thus suppressing the migration and invasion of lung cancer cells. Furthermore, miR-182 inhibited hepatocyte growth factor (HGF)- and phorbol-12,13-dibutyrate (PDBu)-induced invadopodia formation and ECM degradation by inhibiting cortactin. We conclude that miR-182 functions as a tumor suppressor to inhibit cancer metastasis.

## Methods

### Patients and tissue specimens

The study participants included 55 patients, as previously reported [23], who had been diagnosed with NSCLC and had undergone surgical resection of lung cancer between 2006 and 2010 at the Department of Lung Cancer Surgery, Tianjin Medical University General Hospital. Forty two cases presented with lymph node metastasis and 13 cases without lymph node metastasis. Lung cancer staging for each patient was performed according to the AJCC Cancer Staging Manual, 7th edition, and was based on findings from physical examination, surgical resection, and computed tomography of the chest, abdomen, pelvis, and brain. Two cases were stage I, 32 cases were stage II, 13 cases were stage III, and 8 cases were stage IV. Follow-up information was obtained from the medical records of the patients. The following information was collected at the time of diagnosis: sex, age, histology, smoking status, lymph node metastasis status, and clinical stage (Additional file 1: Table S1). Tissue samples were collected from surgically resected tissues and immediately stored in liquid nitrogen until RNA and miRNA extraction.

### Gene expression omnibus(GEO) data sets analysis

The mRNA expression profile of GSE31210 was downloaded from the Gene Expression Omnibus (http://www.ncbi.nlm.nih.gov/geo) based on the platform Affymetrix Human Genome U133 Plus 2.0 Array (Santa Clara, CA, USA). Exploring differentially expressed gene sets between normal and cancer profiles in GSE31210 was performed via the GCBI (https://www.gcbi.com.cn) online tool. CTTN probe expressions were extracted from the above data sets and compared in patient samples stratified by different clinical parameters.

### Cell culture and transient transfection

The A549 cell line was obtained from American Type Culture Collection (Manassas, VA, U.S.A.). The H1299 and 95C cell lines were obtained from the Cell Bank of the Chinese Academy of Sciences (Shanghai, China). All cell lines were maintained at 37 °C in RPMI 1640 medium supplemented with 10% fetal bovine serum(FBS), 100 IU/ml penicillin and 100 μg/ml streptomycin (Invitrogen, Carlsbad, CA, USA). The miR-182 mimics, non-specific control miRNA (scrambled control), the miR-182 inhibitor, small interfering RNA (siRNA) against human CTTN, Cdc42, N-WASP and siRNA negative control were purchased from Riobio (Riobio, Guangzhou, China). For transient transfection, cells were seeded in six-well culture plates and transfection was performed at 60% cell confluence using Lipofectamine 2000 (Invitrogen, Carlsbad, CA, USA) according to the manufacturer’s instructions.

### Wound-healing assay

The migration capacity of cells was measured as reported previously [23]. Briefly, after the growing cells layers reached confluence, a 200 μL sterile pipette tip was used to create an artificial wound. The suspended cells were rinsed and removed with serum-free medium. The initial gap length (0 h) and residual gap length 24–48 h after scratching were observed under an inverted microscope. Experiments were performed in triplicate.

### Transwell migration and invasion assay

In vitro transwell migration and invasion assays were performed, as described previously [23]. Briefly, cells were washed three times with PBS. For migration assays, 5 × 10^4^ cells were added to the upper Transwell chambers (Corning Costar, Corning, NY, USA) in serum-free medium. For invasion assays, 1 × 10^5^ cells in FBS-free medium were plated in the upper chamber coated with Matrigel (Corning Costar, Corning, NY, USA), and 600 μL serum containing 10% FBS medium was added to the lower chamber. After 24 h or 48 h of incubation, cells that had invaded the lower side were fixed with 4% paraformaldehyde and stained with crystal violet solution (Beyotime Biotechnology, China). This was followed by imaging and counting with an IX71 inverted microscope (Olympus, Tokyo, Japan). Five random fields were counted. For the Transwell migration assay, the remaining protocol was the same as the Transwell invasion assay, without pre-coating of the Matrigel in the inserts.

### Western blot analysis

Total protein was prepared with a RIPA buffer (1 × PBS, 1% NP40, 0.5% sodium deoxycholate, 0.1% SDS) supplemented with 2 mM PMSF (Thermo Fisher Scientific, Inc., Waltham, MA, USA). Protein concentrations were measured using a BCA protein assay kit (Pierce Biotechnology, Rockford, IL, USA). Protein extracts (20-30 μg) were denatured with 10% SDS-PAGE, and then transferred to PVDF membranes (Merck Millipore, Billerica, MA, USA). After blocking by incubation in 5% non-fat milk in Tween-TBS (0.1 M, pH 7.4), membranes were incubated with primary antibodies against CTTN (1:400 dilution, Santa Cruz Biotechnology, CA, USA), phosphorylated- (p-) CTTN (1:1000 dilution, Cell Signaling Technology, Inc., Danvers, MA, USA), Rac1 (1:1000 dilution, BD Biosciences, Franklin Lakes, NJ, USA), Rock1 (1:1000 dilution, ProteinTech Group Inc. Chicago, IL, USA), Cdc42 (1:1000 dilution, ProteinTech Group Inc. Chicago, IL, USA), N-WASP (1:1000 dilution, ProteinTech Group Inc. Chicago, IL, USA), Arp2, MT1-MMP, MMP2 and MMP9 (1:1000 dilution, Cell Signaling Technology, Inc., Danvers, MA, USA) and β-actin (1:3000 dilution, Sigma Aldrich, St. Louis, MO, USA) overnight at 4 °C, and then exposed to HRP-conjugated secondary antibody (1:1000 dilution, Thermo Fisher Scientific, Inc., Waltham, MA, USA) for 1 h at room temperature. Bands were visualized using Pierce ECL Substrate (Thermo Fisher Scientific, Inc., Waltham, MA, USA).

### miRNA and mRNA quantitative real-time PCR

Total RNA was extracted from cultured cells or tissues using TRIzol Reagent (Invitrogen, Carlsbad, CA, USA) according to the manufacturer’s instructions. Briefly, 2 μg of extracted RNA was reverse transcribed to cDNA with the application of reverse transcriptase (Promega, Beijing, China). The qRT-PCR reactions were performed with Power SYBR Green PCR Master Mix (Applied Biosystems, Foster, CA, USA), and GAPDH was used as an internal control. Then, miRNAs were extracted from tissues and cell lines using the miRNeasy mini kit (QIAGEN, Cat. 217,004) according to the manufacturer’s instructions. RNA was quantified using a spectrophotometer (Beckman, USA), and RNA quality was assessed using a denaturing 1.2% agarose gel. Finally, miRNAs were quantified by using Bulge-Loop™ miRNA qRT-PCR primer sets (Riobio, Guangzhou, China). U6 was applied as the internal control for target genes. The performance analysis was carried out using the 2^-ΔΔCT^ methods.

### Luciferase assays

The potential miR-182-binding sites in the CTTN 3’UTRwere predicted by TargetScan7.1 (http://www.targetscan.org) and miRanda (http://www.microRNA.org). A sequence containing the presumed miR-182 binding site of the 3’-UTR of CTTN was inserted into the p-miRGLO-reporter plasmid (Promega, Madison, WI, USA). For point mutations, we used the Fast Site-Directed Mutagenesis Kit (Tiangen, Beijing, China) according to the manufacturer’s instruction. HEK-293 cells were seeded into 24-well plates 1 day before transfection, and then co-transfected with 30 ng of wild type or mutated p-miRGLO-CTTN vectors and 50 nM of miR-182 mimics or scrambled control (Riobio, Guangzhou, China). After 48 h, the luciferase activities were determined with a dual-luciferase reporter assay kit (Promega, Madison, WI, USA) according to the manufacturer’s instructions.

### Immunoflourescence assay

Cells cultured on cover glass slides were fixed with 4% paraformaldehyde at room temperature for 30 min. Cells were then permeabilized in 0.5% Triton X-100 for 15 min and blocked in PBS containing 1% BSA for 60 min at room temperature. Cells were then incubated with an anti-cortactin antibody (Santa Cruz Biotechnology, CA, USA) at a dilution of 1: 200 at 4 °C overnight, followed by incubation with Alexa Fluor-conjugated secondary antibodies or phalloidin for 1 h each at room temperature. After washing with PBS, DAPI (Sigma Aldrich, St. Louis, MO, USA) was added for 5 min and images were taken using an inverted fluorescence microscope (Olympus IX7, Tokyo, Japan).

#### Statistical analyses

All data analyses were performed using SPSS 21.0 (IBM Corp., Armonk, NY, USA). Data are presented as mean ± standard deviation(SD). Statistical analyses were performed using a paired Student’s t-test or one-way analysis of variance (ANOVA). The correction between cortactin expression and metastases in primary lung cancer was analyzed by the Wilcoxon two-sample test. The Kaplan–Meier method was used to estimate overall survival (OS) and progression-free survival (PFS). A *P*-value < 0.05 indicates statistical significance.

## Results

### Cortactin is upregulated in lung cancer tissues and is a marker of poorer prognosis in human NSCLC

**Fig. 1:**
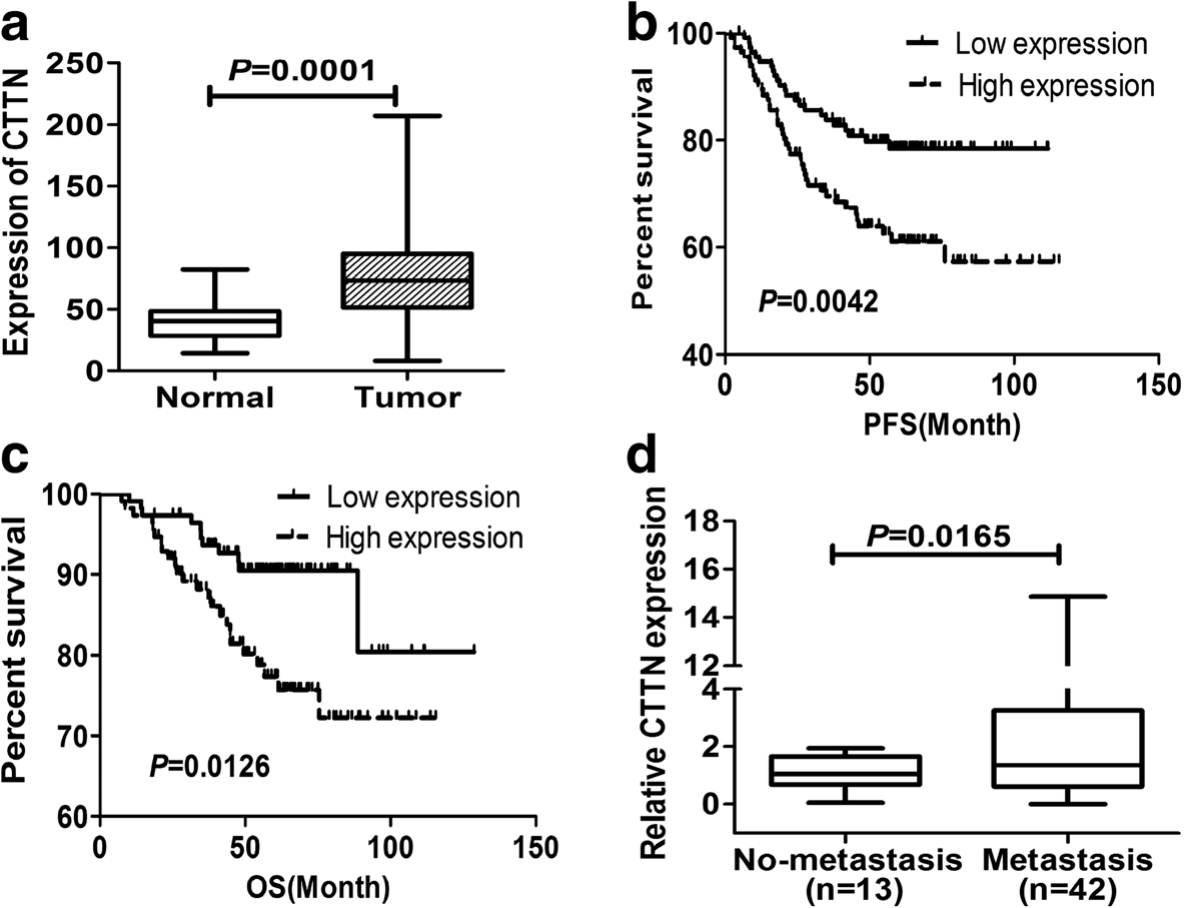
Cortactin was upregulated in lung cancer tissues and could be a marker of poorer prognosis in human NSCLC. **a** Expression profiles of CTTN in primary lung cancer and matched normal tissues were reported as 2^-∆Ct^ values. **b** and **c** Kaplan-Meier curves for progression-free survival (PFS) and overall survival (OS) according to expression levels of cortactin. PFS and OS rate were calculated by log-rank test. **d** Cortactin expression in lung cancer tissues was quantified as 2-^∆Ct^ values. Cortactin expression levels in primary metastasis-positive NSCLC tissues were compared with metastasis-negative tissues

Cortactin(CTTN) is known to be over-expressed in multiple solid tumors [9–11]. To determine its expression level in NSCLC compared to non-cancerous adjacent lung tissues, the GSE31210 dataset that included 226 lung adenocarcinoma samples from the GEO database was analyzed. Of these patients, 168 were stage I and 58 were stage II. This dataset also contained 20 adjacent normal lung tissue samples. RNA expression data from these databases demonstrated that cortactin was expressed in lung cancer tissues 2.4-fold more than non-cancerous adjacent lung tissues (Fig. 1a). To investigate the correlation between cortactin expression and the prognosis of NSCLC patients, Kaplan-Meier survival analysis was performed. Results demonstrated that the high level of cortactin expression predicted a shorter PFS (log rank =8.201, *P* = 0.0042) and OS (log rank =6.225, *P* = 0.0126) in lung cancer patients (Fig. 1b and c). These data imply that overexpression of cortactin contributes to progression and poor prognosis of NSCLC.

To further examine the correlation between expression of cortactin and metastasis in NSCLC tissue samples, we detected the expression of CTTN in 55 human NSCLC tissues by qRT-PCR. As shown in Fig. 1d, the mean expression of cortactin was significantly higher in the group of patients with lymph node metastases (42/55 cases) compared to those without lymph node metastases (13/55 cases) (Wilcoxon two-sample test, *r*_s_ = 0.1076, *P* = 0.0165). These data further confirmed that the higher expression of CTTN contributed to the metastasis of NSCLC.

### Cortactin promotes the migratory and invasive potential of NSCLC cells in vitro

**Fig. 2:**
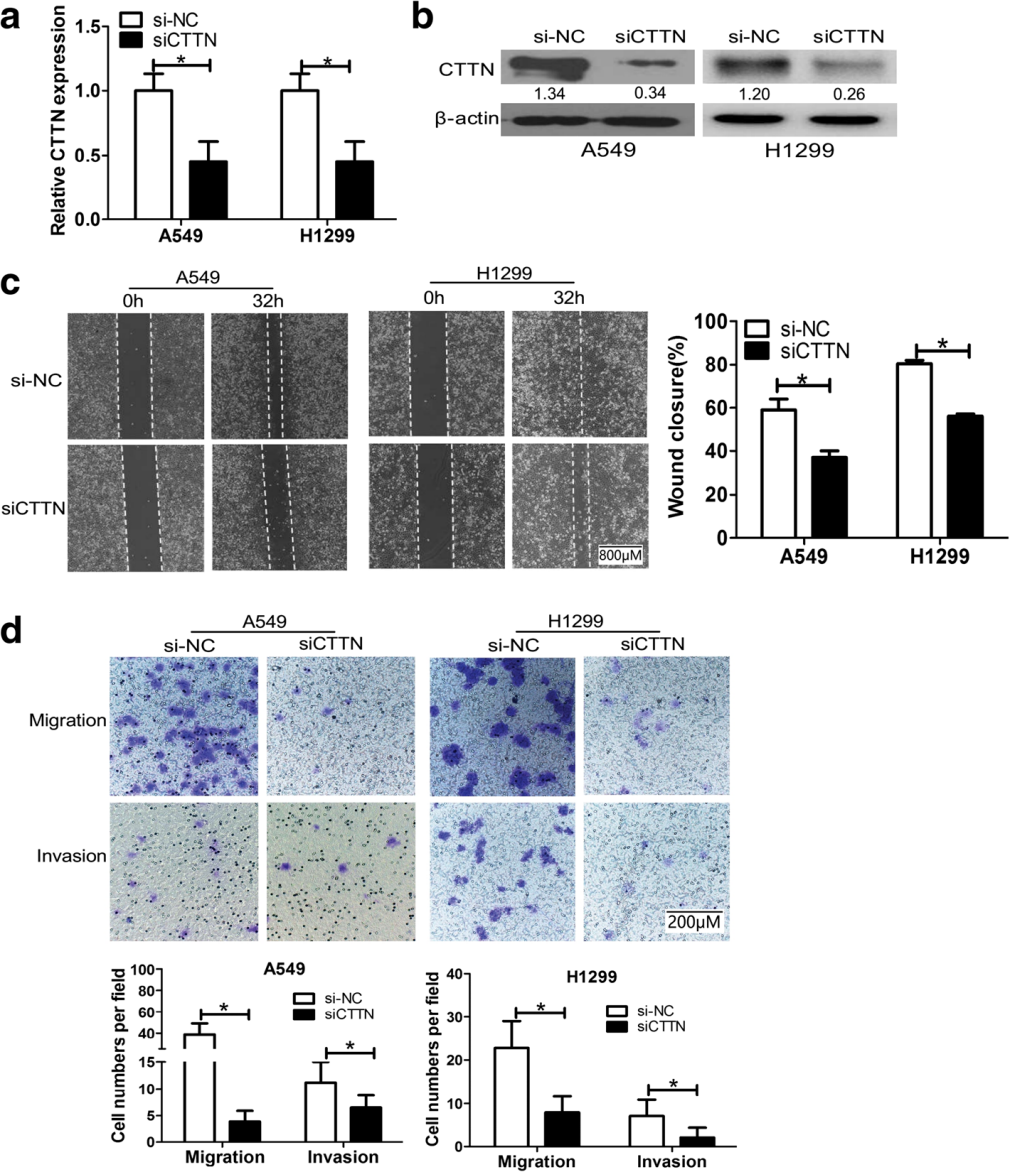
Cortactin promoted the migratory and invasive potential of NSCLC cells in vitro. **a** A549 and H1299 cells were transfected with cortactin small interfering RNA (siRNA) or the siRNA control oligonucleotides, followed by qRT-PCR analysis of cortactin levels in the differently treated cells. **b** Protein expression levels of cortactin were measured by Western blotting. **c** Wound healing assay in A549 and H1299 cells transfected with cortactin siRNA or siRNA control oligonucleotides. After 32 h, wound healing assays were performed in triplicate. Representative images are displayed Scale bar, 800 μm, 4× magnification. The values shown are the mean ± SD, *P < 0.05. **d** Transwell migration and invasion assays of A549 and H1299 cells were performed after transfection with cortactin siRNA or siRNA control oligonucleotides. Representative figures of the migrated and invaded stained cells are shown on the left. Data are shown as the mean ± SD. Representative images are displayed on the right. Scale bar, 200 μm, 20× magnification, *P < 0.05

Cortactin is an important regulator involved in the invasion and migration of cancer cells [12, 13]. The fact prompted us to investigate the influence of cortactin on the malignant phenotypes in lung cancer cells. To unravel the function of cortactin in the metastasis of NSCLC, cortactin was significantly downregulated in A549 and H1299 cells by siRNA (CTTN -siRNA). Both qRT-PCR and western blot analysis revealed that CTTN expression was effectively knocked down by siRNA interference (Fig. 2a-b). At 48 h after cortactin-siRNA transfection, cell migration and invasion were observed in A549 and H1299 cells by wound healing assays and Boyden chamber assays. The wound healing assays showed that silencing of cortactin expression significantly inhibited the migration capacity in both A549 and H1299 cells (58.18% versus 35.74 and 77.66% versus 55.14%, *P* = 0.015 and *P* = 0.007, respectively, Fig. 2c). Consistent with these results, we found that CTTN siRNA transfected cells showed a significant decrease in the number of migrated and invaded cells in A549 and H1299 cells compared with the scrambled control transfection (39.2 versus 2.4, *P* = 0.008 and 22.4 versus 6.8, *P* = 0.021 for migration, respectively; 9.8 versus 5.8, *P* = 0.024 and 6.0 versus 2.2, *P* = 0.034 for invasion, respectively, Fig. 2d).These results proved that cortactin promotes migration and invasion in NSCLC.

### PDBu and HGF induce invadopodia formation, cell migration and cortactin expression in NSCLC

**Fig. 3:**
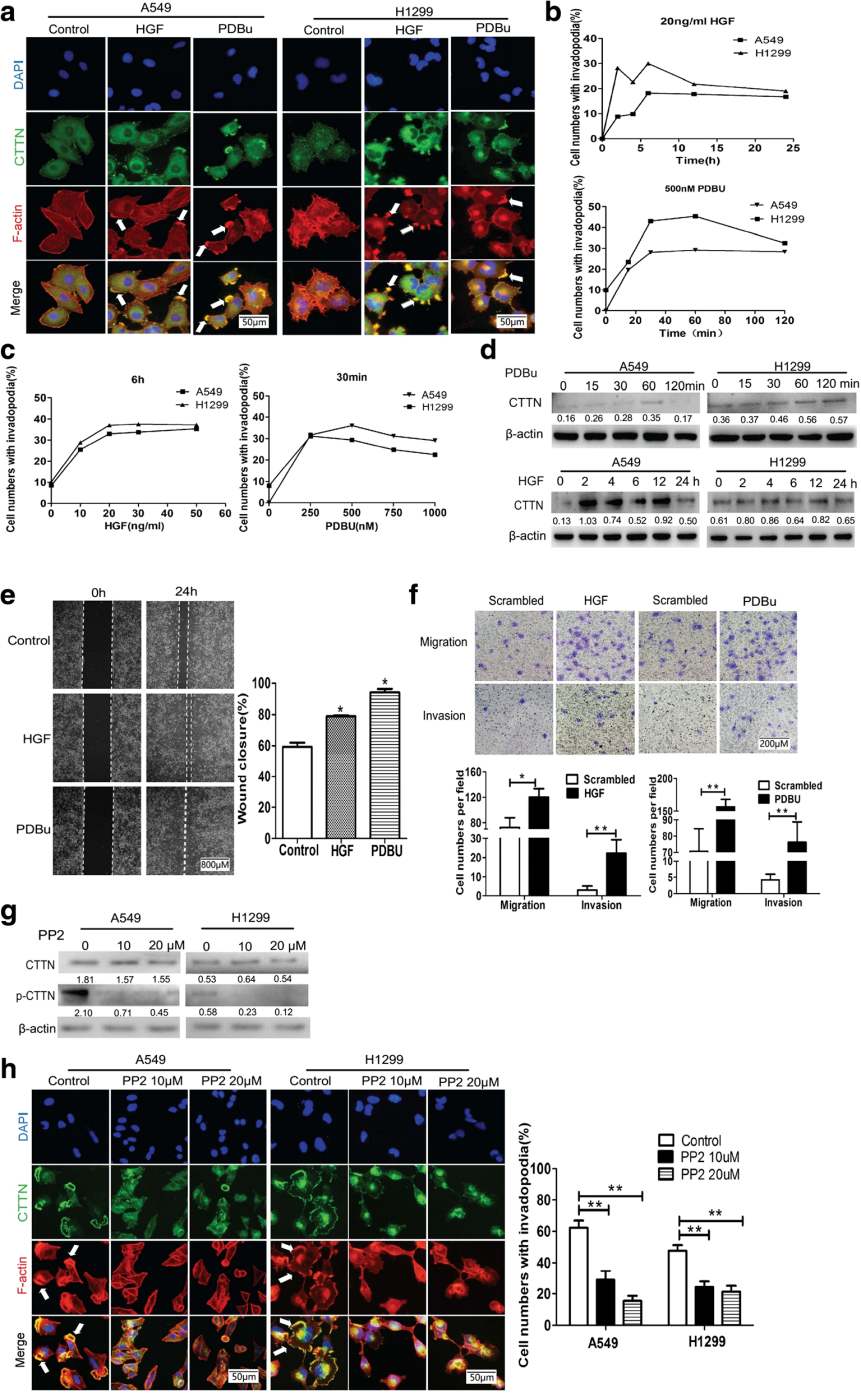
PDBu and HGF promoted cortactin expression, cell migration and invadopodia formation in NSCLC. **a** A549 and H1299 cells were treated with or without HGF (20 ng/mL) or PDBu (500 nM), and invadopodia were visualized by colocalization of cortactin staining with FITC (green), F-actin with phalloidin (red) and nuclei with DAPI (blue). representative images are displayed. Scale bar, 50 μm, 40× magnification. **b** Quantification of cells with invadopodia. **c** A549 and H1299 cells treated with different concentrations of HGF for 6 h or PDBu for 30 min; Cells with invadopodia were quantified. **d** Cell lysates from A549 and H1299 cells treated with 20 ng/mL HGF or 500 nM PDBu after blotting with cortactin antibody. **e** Wound healing assays in A549 cells were performed after treatment with 20 ng/mL HGF for 6 h or 500 nM PDBu for 30 min. Representative images are displayed. Scale bar,800 μm, 4× magnification, *P < 0.05. **f** Transwell migration and invasion assays of A549 cells were performed after treatment with 20 ng/mL HGF for 6 h or 500 nM PDBu for 30 min. Representative figures of the migrated and invaded stained cells are shown. Scale bar, 200 μm, 20× magnification. Data are shown as the mean ± SD, *P < 0.05, **P < 0.01. **g** Following treatment with 0, 10, and 20 μM PP2 for 24 h, A549 and H1299 cells were harvested. Levels of CTTN and the phosphorylation of CTTN were determined using Western blot analysis. **h** A549 and H1299 cells treated with 10 μm PP2 or 20 μm PP2 for 24 h, invadopodia were visualized as above. Representative images are displayed. Scale bar, 50 μm, 40× magnification, **P < 0.01

Invadopodia formation is thought to be essential for invasion and metastasis. Emerging evidence has revealed a critical role for cortactin in invadopodia formation as well as in promoting cell motility and invasion [6–8]. Many stimuli, including PDBu and HGF, have been reported to induce invadopodia formation in specific cell types [24–26]. PDBu and HGF also function as potent stimulators of cell migration in cancer [27, 28]. To determine the effects of PDBu and HGF on invadopodia formation, as well as investigating the expression of cortactin in lung cancer cells, A549 and H1299 cells were stimulated with PDBu or HGF, or were unstimulated, and the changes in cell morphology and invadopodia were recorded with immunofluorescence assays. Cortactin (green) was redistributed to newly-formed invadopodia in the cytoplasm and ruffles at the edges of the cytoplasmic membrane, Filamentous (F)-actin was stained with phalloidin and cortactin co-localized with F-actin (red) in the newly-formed invadopodia of the cells. Untreated cells displayed longer and thinner stress fibers in the cytoplasm, and cortical filaments along the periphery. After 500 nM PDBu or 20 ng/ml HGF treatment, cell morphology changed rapidly, inducing dramatic rearrangement of F-actin structures. The newly-formed invadopodia were characterized as actin-rich small dots, rosette rings, and belts in the cytoplasm, and membrane ruffles at the edge of the peripheral membrane (Fig. 3a). The newly-formed cytoskeletal structures appeared within 20 min when the cells were treated with 500 nM of PDBu or within 2 h when treated with 20 ng/ml HGF. Moreover, and the number of newly-formed invadopodia peaked at 60 min after PDBu treatment and at 6 h after HGF treatment(Fig. 3b). We also found that the number of newly-formed invadopodia were peaked at 20 ng/ml when treated with HGF for 6 h or 500 nM when treated with PDBu for 30 min (Fig. 3c).

To determine whether PDBu or HGF could affect the cortactin expression, we tested cortactin expression and the cell migration and invasion ability in PDBu- or HGF-induced lung cancer cells. The results showed that cortactin expression was induced and peaked at 60–120 min after PDBu treatment, and peaked between6-12 h in the HGF-treated lung cancer cells (Fig. 3d). Meanwhile, the impact of PDBu or HGF on cell migration and invasion ability was also evaluated. Wound healing assays and boyden chamber transwell assays were performed in A549 cells after treatment with PDBu or HGF. As shown in Fig. 3e, the wounded area of the PDBu- or HGF-treated groups was significantly decreased compared with that in the control group (59.24% versus 78.88 and 94.04%, *P* = 0.00099 and *P* = 0.00013, respectively, Fig. 3e), and a significantly increased number of migrated and invaded cells were observed when cells were treated with PDBu or HGF, compared with the control group. PDBu or HGF treatment resulted in an increased cell motility in A549 cells (72.8 versus 120.2, *P* = 0.0016 for HGF, and 3.0 versus 22.2, *P* = 0.0005 for PDBu, respectively, Fig. 3f). Consistent with these results, the Transwell assay with Matrigel suggested that PDBu or HGF could induce invasion of lung cancer cells (70.6 versus 156.6, *P* = 0.0001 for HGF, and 4.2 versus 76.2, *P* < 0.0001 for PDBu, Fig. 3f).

Cortactin is phosphorylated by many different kinases, and the phosphorylation status of CTTN were found to be important for invasive functions such as invadopodia-associated ECM degradation and experimental metastasis. Many different pharmaceuticals, including PP2, which is a Src inhibitor, were reported to inhibit the phosphorylation of cortactin and inhibit the formation of invadopodia [29]. To further address the impact of CTTN phosphorylation on invadopodia, we treated A549 cells with PP2. We found that phosphorylation of CTTN at A549 was significantly reduced by PP2, while the total levels of CTTN are unaffected (Fig. 3g). Furthermore, as showed in Fig. 3h, the percentage of invadopodia cells induced by PDBu was significantly decreased by 10 μM or 20 μM PP2 treatment (62.2% versus 28.7 and 15.4%, *P* = 0.0056 and *P* = 0.0008 for A549, respectively; 47.6% versus 24.2 and 21.2%, *P* = 0.0018 and *P* = 0.0017 for H1299, respectively).These data strongly support an important role for cortactin phosphorylation in promoting invadopodia .

These observations indicated that PDBu and HGF could induce invadopodia formation, as well as cortactin expression and stimulate cell migration in non-small cell lung cancer cells.

### Cortactin is a direct target of miR-182 in NSCLC cells

**Fig. 4:**
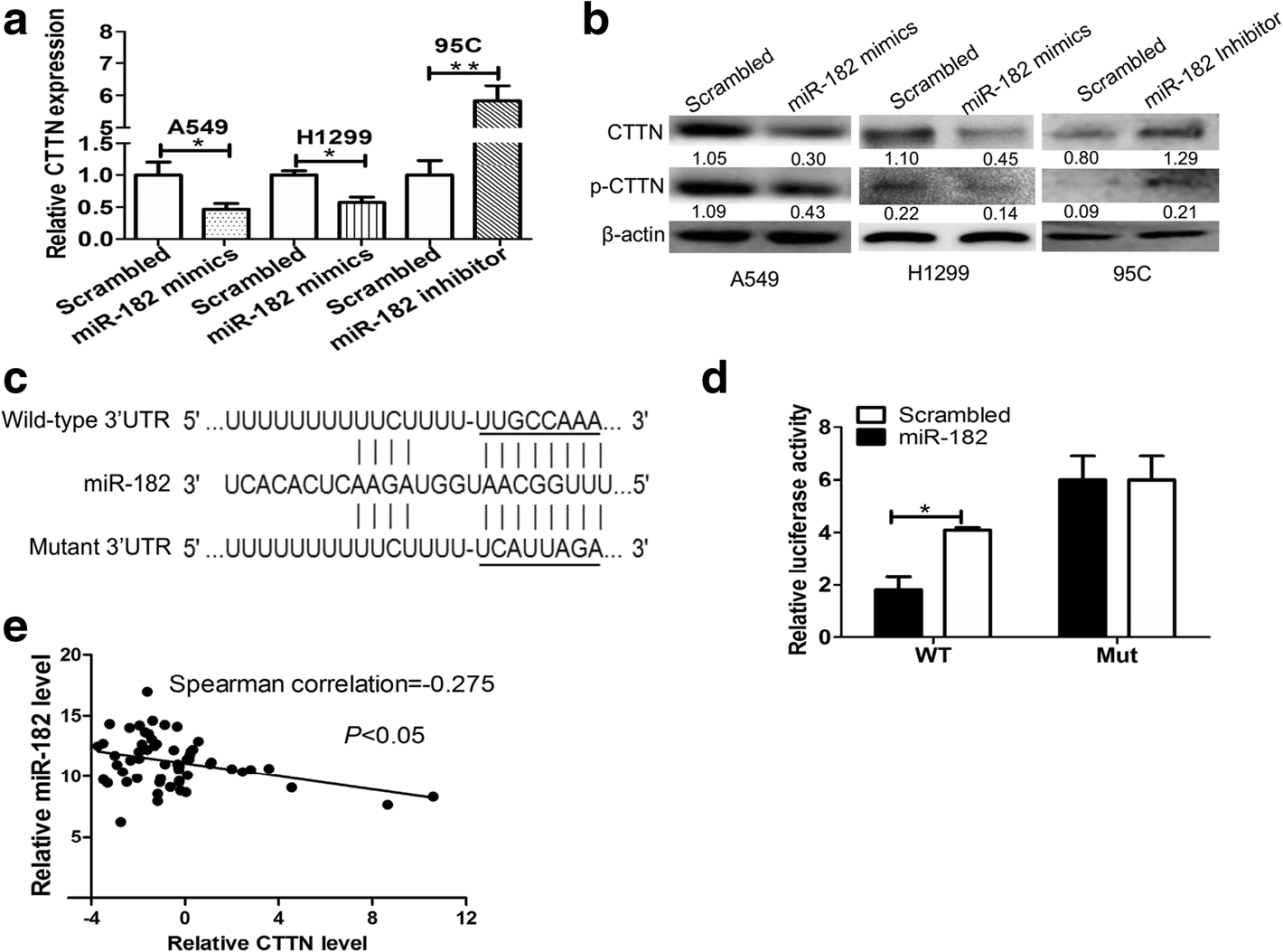
Cortactin was a direct target for miR-182 in NSCLC cells. The expression levels of cortactin mRNA and protein were down-regulated when transfected with miR-182 mimics in A549 and H1299 cells, while cortactin mRNA and protein expression in 95C cells were up-regulated when treated with miR-182 inhibitor; these were shown using (**a**) real-time PCR assays and (**b**)Western blotting, respectively. *P < 0.05 and **P < 0.01. **c** Predicted miR-182 target sequence in the 3’-UTR of CTTN mRNA. The mutant binding site is underlined. **d** Luciferase reporter assay of HEK-293 cells co-transfected with wild-type or mutant cortactin3’-UTR reporter plasmid and miR-182 or scrambled miRNA. *P < 0.05 (**e**) The level of miR-182 was inversely correlated with cortactin expression in human NSCLC tissues. Cortactin and miR-182 expression levels were detected by qRT-PCR

Accumulating studies have suggested that miRNAs play critical roles in the development and progression of non-small cell lung cancer (NSCLC) [30]. Recently, Zhang et al. showed that miR-182 could regulate human cortical actin-associated protein and inhibit the proliferation and invasion of human lung adenocarcinoma cells [31]. By using several computational methods and predictions by both the TargetScan60 and miRanda search programs, it was shown that cortactin is a potential direct target of miR-182. To confirm whether cortactin was a direct target of miR-182, we first transfected the A549, H1299 and 95C cells with miR-182 mimics or an inhibitor, or a scrambled negative control for 48 h, the mRNA and protein expressions of cortactin were measured by qRT-PCR and Western blotting, respectively. Data demonstrated that overexpression of miR-182 attenuated cortactin mRNA expression in lung cancer cells. Conversely, inhibition of miR-182enhanced cortactin mRNA expression in 95C cells (Fig. 4a). Consistent with the above results, we found that the protein expression of cortactin was dramatically decreased in A549 and H1299 cells when transfected with miR-182 mimics, while miR-182 inhibitor increased the cortactin expression in 95C cells (Fig. 4b). It is reported that cortactin is activated via phosphorylation, regulating cell motility, rac1-mediated actin dynamics, cadherin-dependent adhesion, chemokine trafficking and chemokine-dependent chemotaxis [7, 8]. Here we also detect the phosphorylation of cortactin when miR-182 expression changed. Our data showed that the phosphorylation level of cortactin decreased by miR-182 mimics but increased by miR-182 inhibitor (Fig. 4b).

To investigate the potential mechanisms of miR-182 and cortactin, we then cloned the cortactin 3’-UTR of wild-type or mutant miR-182 target sequences into the pmirGLO vector (Fig. 4c). A luciferase reporter assay was carried out to determine whether miR-182 directly targets the 3’-UTRs of cortactin. Through co-transfection of miR-182 mimics or scrambled negative control in HEK-293 cells, miR-182 significantly decreased the relative luciferase activity of wild-type cortactin 3’UTR, whereas that of the mutant cortactin 3’-UTR did not change significantly, which suggests that CTTN was the target of miR-182 (Fig. 4d). To further explore the relationship between miR-182 and CTTN, we detected CTTN expression levels in 55 pairs of NSCLC tissues. Our qRT-PCR results showed that cortactin expression was inversely correlated with miR-182 expression (Spearman correlation = − 0.275, 95%CI = − 0.5038 to − 0.01007, *P* = 0.0367, Fig. 4e). These results showed that CTTN may be a direct downstream target for miR-182.

### miR-182 suppressed PDBu- and HGF-induced invadopodia formation and metastasis of lung cancer cells by regulating cortactin

**Fig. 5:**
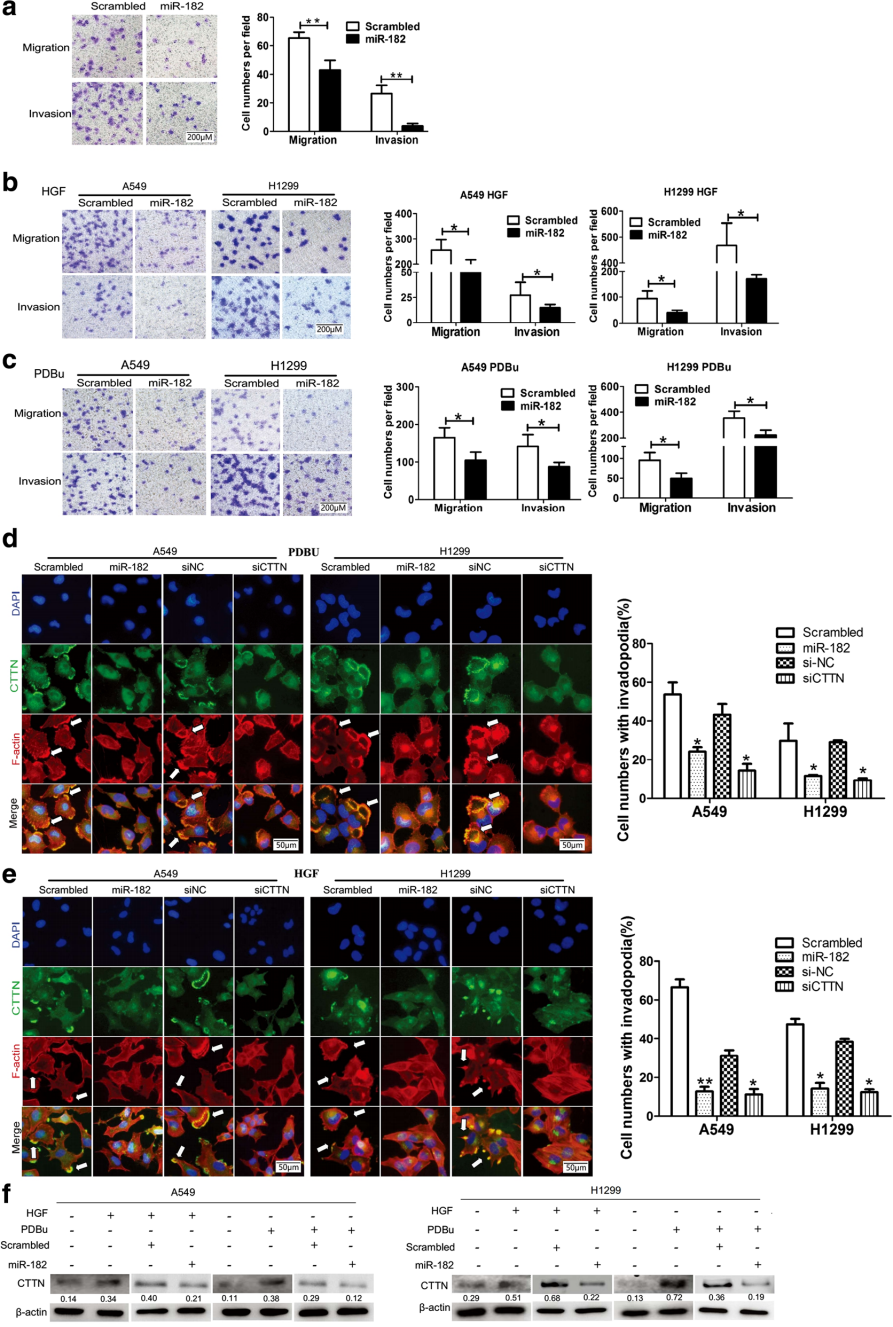
miR-182 suppressed PDBu- and HGF-induced invadopodia formation and metastasis of lung cancer cells by regulating cortactin. **a** Transwell analysis of migrated and invaded A549 cells treated with miR-182 or scrambled miRNA. Representative images are displayed. Scale bar, 200 μm, 20× magnification, **P < 0.01. **b** and **c** A549 and H1299 cells were transfected with miR-182 or scrambled miRNA and then subjected to transwell assay in the presence of HGF treatment for 6 h or PDBu treatment for 30 min. Representative figures of the migrated and invaded stained cells are shown on the left. Data are shown as the mean ± SD. Scale bar, 200 μm, 20× magnification, *P < 0.05. **d** and **e** A549 and H1299 cells were transfected with scrambled miRNA, miR-182, si-NC or CTTN siRNA in the presence of (**d**) PDBu treatment for 30 min or (E) HGF treatment for 6 h, and stained for cortactin (green), F-actin (red), and nuclei (blue). Cells with invadopodia were quantified, and representative images are displayed on the right. Data are shown as the mean ± SD. Scale bar, 50 μm, 40× magnification, *P < 0.05. **f** After treatment with PDBu or HGF, protein expression of CTTN in A549 and H1299 cells transfected with scrambled miRNA or miR-182 were analyzed by SDS-PAGE. β-actin was used as a control

To explore the role of miR-182 in NSCLC, lung cancer cell lines were transfected with miR-182 mimics and further used for functional analysis. It was shown that miR-182 markedly suppressed the migration and invasion of A549 cell lines (65.2 versus 43.0, *P* = 0.0001 for migration, and 26.5 versus 3.8, *P* = 0.0005 for invasion, respectively, Fig. 5a), which was also reported in our previous work [32]. At the same time, miR-182 transfection reduced the migration and invasion ability induced by PDBu and HGF, which was detected by transwell assays (Fig. 5b-c). To further investigate whether miR-182 can regulate the formation of PDBu- and HGF-induced invadopodia formation in lung cancer cells, an immunofluorescence assay was performed to detect invadopodia changes after treatment with PDBu or HGF. As shown in Fig. 5d and e, ectopic expression of miR-182 highly reduced the number of invadopodia induced by PDBu (71.0% versus 42.2%, *P* = 0.0174 A549; 24.2% versus 2.8%, *P* = 0.001 for H1299, respectively) compared to the scrambled control. Similar results were obtained in experiments when cells were treated with HGF (32.6% versus 21.8%, *P* = 0.021 for A549; 36.8% versus 25.4%, *P* = 0.031 for H1299, respectively). Moreover, silencing expression of cortactin could result in reduction of invadopodia, which induced by PDBu or HGF (Fig. 5d and e). Western blot assays were then performed to investigate whether the protein expression of cortactin was influenced by miR-182 in the cells treated with PDBu or HGF. Compared to the control cells, cortactin protein was up-regulated in cells with PDBu or HGF, but partly recuperated by miR-182 mimics (Fig. 5f). These results indicate that miR-182 could inhibit the formation of PDBu- and HGF-induced invadopodia and metastasis of lung cancer cells by targeting cortactin.

### miR-182suppresses invadopodia formation of lung cancer cells via the Cdc42/N-WASP pathway, as well as reduces the Rac1 and Rock1 activities

**Fig. 6:**
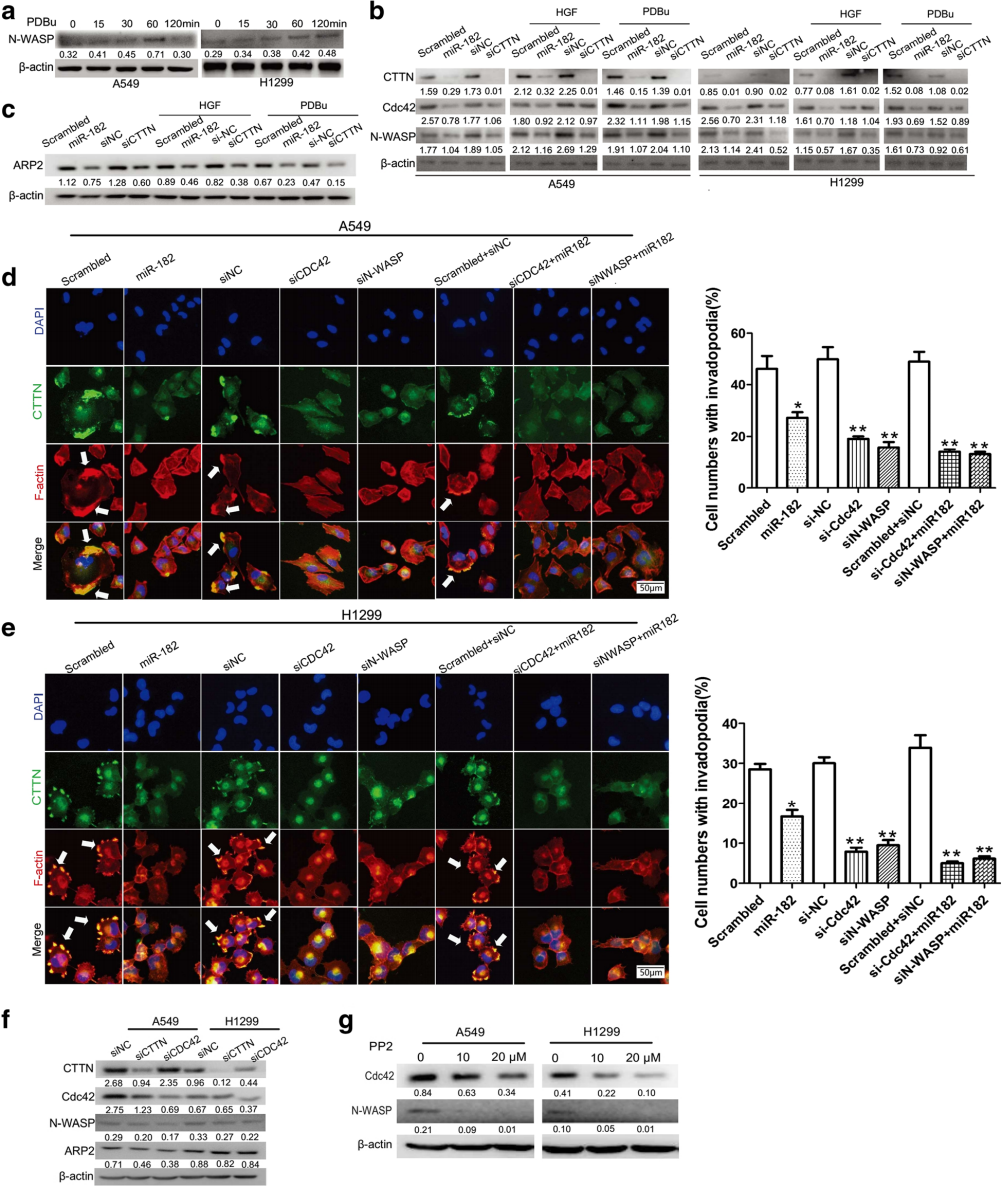
miR-182 suppresses invadopodia formation of lung cancer cells via suppression of the Cdc42/N-WASP pathway. **a** A549 and H1299 cells were pretreated with 500 mM of PDBu for 0 min, 15 min, 30 min, 60 min and 120 min, and then N-WASP protein expression were measured by Western blotting. **b** A549 and H1299 cells were transfected with scrambled miRNA, miR-182, si-NC or CTTN siRNA with or without PDBu or HGF treatment, then subjected to analysis of CTTN, CDC42 and N-WASP expression by Western blotting. **c** Arp2 expression in A549 cells treated with or without HGF or PDBu was detected by immunoblotting. β-actin was used as a control. **d** and **e** A549 and H1299 cells were transfected with scrambled miRNA, miR-182, si-NC, CTTN siRNA, or N-WASP siRNA was co-transfected with miR-182 in the presence of PDBu treatment for 30 min, and stained for cortactin(green), F-actin (red), and nuclei (blue). Cells with invadopodia were quantified. Representative images are displayed on the right. Data are shown as the mean ± SD. Scale bar, 50 μm, 40× magnification, *P < 0.05, **P < 0.01. **f** A549 and H1299 cells were transfected with si-NC, CTTN siRNA or Cdc42 siRNA, then cortactin, Cdc42, N-WASP and Arp2 protein expression were measured by Western blotting. β-actin was used as a control. **g** Following treatment with 0,10, and 20 μM PP2, A549 and H1299 cells were harvested. Levels of Cdc42, and N-WASP, and the phosphorylation of cortactin were determined using Western blot analysis. β-actin was used as a control

The aforementioned study showed that miR-182 directly targets cortactin in lung cancer cells [31]. We then explored the molecular mechanisms responsible for invadopodia formation and the metastasis-suppressive effect of miR-182 in lung cancer cells. Cortactin together with WASP/N-WASP, activates Arp2/3 and induces actin filament assembly and polymerization, and thus induces invadopodia formation and cell migration [3]. As a key invadopodia protein, N-WASP is also a cortactin-binding partner, and binds and recruits the Arp2/3 complex, thus stimulating cortical actin polymerization and invadopodia formation. As shown from our results, both PDBu and HGF treatment induced increased expression of N-WASP (Fig. 6a). However, neither PDBu nor HGF were shown to upregulate N-WASP expression in cortactin-knockdown or miR-182 overexpressed lung cancer cells (Fig. 6b).The cell division cycle protein Cdc42, as a member of the Rho GTPase family, plays an important role in actin polymerization, and was necessary for WASP/N-WASP activation [33]. Western blot analysis showed that Cdc42 protein expression significantly decreased in A549 and H1299 cells transfected with miR-182 mimic or siCTTN in spite of PDBu or HGF induction (Fig. 6b). Furthermore, neither PDBu nor HGF activated Arp2/3 when A549 cells were transfected with miR-182 or siCTTN (Fig. 6c). It was found that knockdown of CDC42 or N-WASP significantly decreased the percentage of invadopodia cells induced by PDBu, which was consistent with the results of the overexpression of miR-182 in lung cancer cells (Fig. 6d and e). We also examined the effect of cortactin siRNA or Cdc42 siRNA on Cdc42, N-WASP and ARP2 expression levels. Remarkably, cortactin silencing decreased expression of Cdc42 in A549 and H1299 cells. Moreover, relatively lower level of N-WASP and ARP2 were detected in the cortactin or Cdc42 siRNA groups than in the untreated ones (Fig. 6f). Similarly, we found the levels of Cdc42 and N-WASP were inhibited byPP2 in A549 and H1299 cells (Fig. 5g). Together, we believe that Cdc42/N-WASP signaling axis was the important downstream target of cortactin in NSCLC. Additionally, miR-182 suppressed the Cdc42/N-WASP pathway, and then suppressed the activation of Arp2/3 via inhibition of cortactin.

**Fig. 7:**
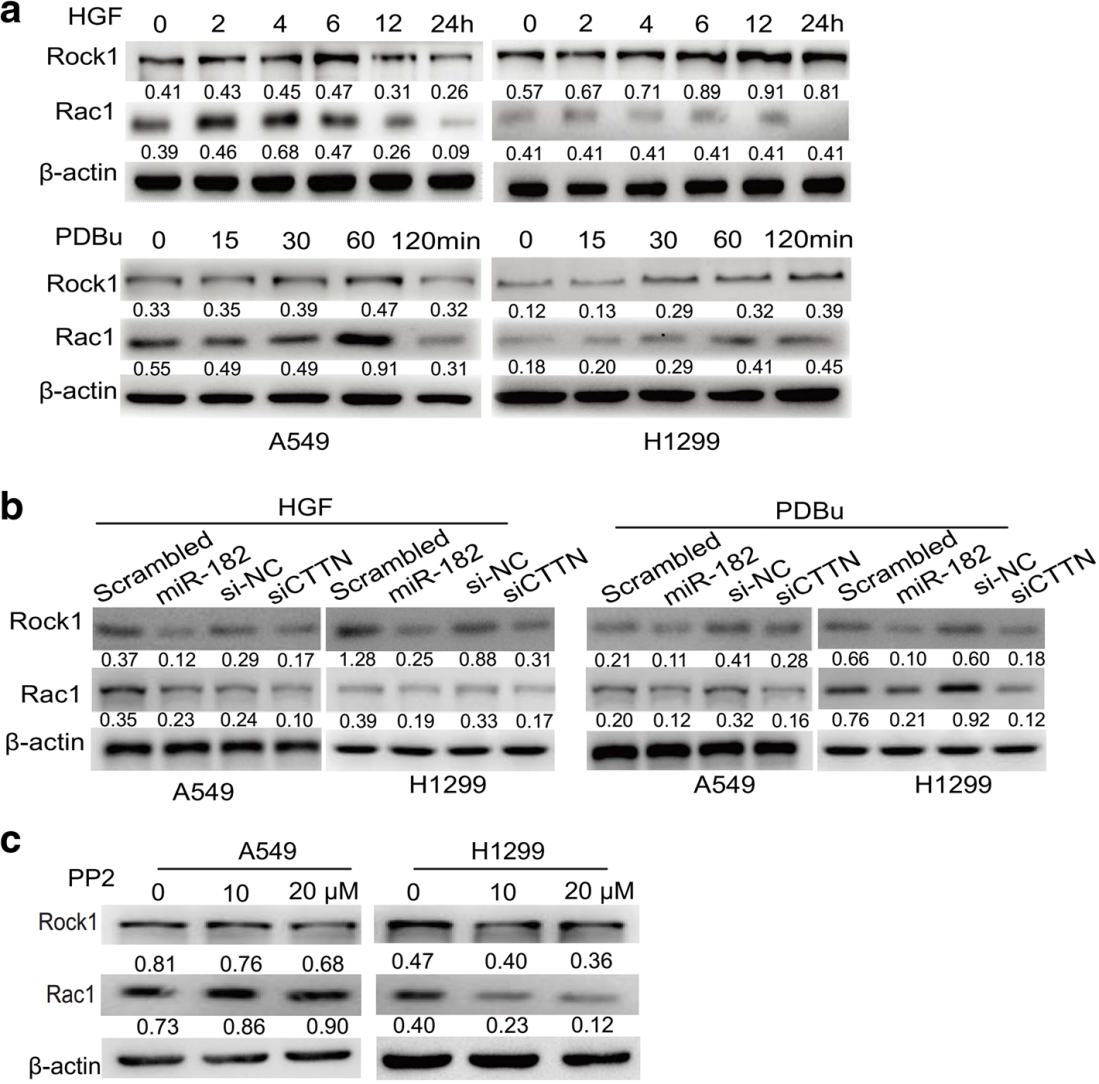
miR-182 inhibited the expression of Rock1 and Rac1 expression in NSCLC cells. **a** Rock1 and Rac1 expression was examined by Western blotting. A549 and H1299 cells were stimulated with 20 ng/mL HGF or 500 nM PDBu for the indicated time before cells were collected and lysed. β-Actin was used as a control. **b** A549 and H1299 cells were transfected with scrambled miRNA, miR-182, si-NC and CTTN siRNA, followed by treatment with or without HGF or PDBu. Cells were then subjected to western blot analysis to examine Rock1 and Rac1 levels. **c** Following treatment with 0,10, and 20 μM PP2, A549 and H1299 cells were harvested. Levels of Rock1 and Rac1 were determined using Western blot analysis. β-actin was used as a control

Rac is an important member of the small GTPase Rho family, which has been shown to regulate many aspects of intracellular actin dynamics [34]. Rac1 can induce F-actin to localize to the cell membrane, where it simultaneously binds F-actin and Arp2/3 to stimulate actin polymerization for invadopodia formation. Asa downstream effector protein of the small GTPase Rho, Rock1 is one of the major regulators of the cytoskeleton [35]. To investigate whether miR-182/CTTN is involved in regulating Rac1 and ROCK by modulating invadopodia formation in lung cancer cells, we measured the protein levels of Rac1 and Rock1. Both PDBu and HGF induced steadily increasing expression of Rac1 and Rock1in A549 cells as well as H1299 cells (Fig. 7a). However, neither PDBu nor HGF upregulated N-WASP expression in CTTN-knockdown or miR-182 overexpressed lung cancer cells (Fig. 7b). We also examined the effect of PP2 on Rac1 and Rock1pression levels. Rac1 and Rock1 levels were significantly decreased after treatment with PP2 in A549 and H1299 cells (Fig. 7c). Overall, these data underscore the importance of cortactin on Rac1 and Rock1activities in NSCLC.

Taken together, these findings indicate that miR-182 suppressed the Cdc42/N-WASP pathway, and then suppressed the activation of Arp2/3 via inhibition of cortactin. As a result, the formation of invadopodia in NSCLC cells was suppressed.

### miR-182 negatively regulates invadopodia function and ECM degradation in lung cancer cells by inhibiting cortactin

**Fig. 8:**
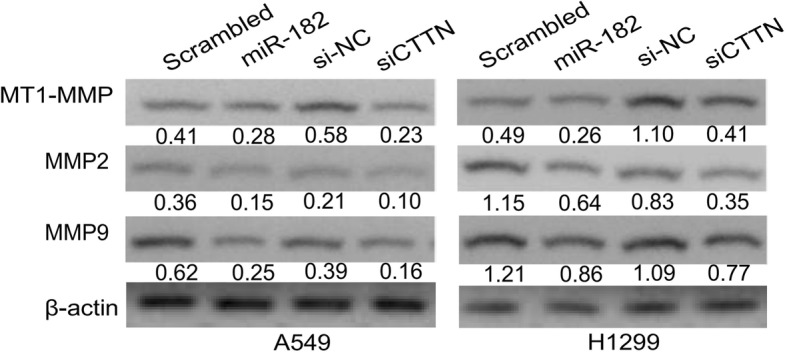
miR-182 negatively regulated invadopodia function and extracellular matrix degradation in lung cancer cells by inhibiting cortactin. The protein expression of MT1-MMP, MMP2 and MMP9 in A549 and H1299 cells transfected with scrambled miRNA, miR-182, si-NC, and CTTN siRNA was analyzed by Western blotting. β-actin was used as a control

Invadopodia are involved in ECM proteolysis, allowing tumor cells to infiltrate the stroma and vasculature. Maturation of pre-invadopodia involves recruitment and activation of matrix metalloproteinase MMP-14 (MT1-MMP) to initiate ECM degradation. MT1-MMP localizes exclusively at the invadopodia edge, which degrades a number of ECM macromolecules and clears a path for tumor cell migration [36, 37]. To gain further insight into the effect of miR-182 on the function of invadopodia, we examine whether miR-182 would affect MT1-MMP in NSCLS. Western blot for MT1-MMP showed that both miR-182 and siCTTN down-regulated MT1-MMP expression in both A549 and H1299 cells. In addition, miR-182 and siCTTN caused downregulation of MMP2 and MMP9, which are two matrix metalloproteinases that degrade type IV collagen, the major structural component of basement membrane (Fig. 8).

## Discussion

It is well known that miRNAs function as critical regulators of gene expression in various biological processes, including cell proliferation, cellular apoptosis, signal transduction and carcinogenesis. As a member of the miR-183/− 96/− 182 cluster located on 7q31–34, miR-182 has been shown to be an important player in regulating tumor progression. However, the roles of miR-182 in different types of tumors are varied and sometimes contradictory. For example, some studies have reported that miR-182 may function as an oncomiR in several tumors [21, 22, 38]. However, microRNA-182 was reported to be a tumor suppressor in other studies [31, 39]. Taken together, these results suggest a highly complex mechanism of miR-182-related tumorigenesis. Here, we clarify the role of miR-182 in human NSCLC metastasis. Our findings showed that miR-182 is a metastasis suppressor gene, and inhibited invadopodia formation and function, thus suppressing metastasis of lung cancer cells by targeting cortactin.

Metastasis is the leading cause of cancer-related deaths, and invadopodia play a key role in promoting metastasis. Cortactin, an important molecule for invadopodia, is frequently used as an invadopodia marker, and is also a universally important player in their function. Firstly, cortactin may act directly to promote actin assembly at invadopodia puncta. Secondly, many key invadopodia proteins are also cortactin-binding partners, including N-WASP, WIP, dynamin, ASAP1/AMAP1, and Src kinase [40], suggesting that cortactin also acts as a scaffolding function in invadopodia. In addition, cortactin was also reported to be an essential regulator of matrix metalloproteinase secretion and ECM degradation in invadopodia. Ultimately, cortactin is a critical mediator of tumor cell invasion. Cortactin is frequently overexpressed in many types of human cancers, including head and neck and esophageal squamous carcinomas, colorectal, gastric, hepatocellular, breast and ovarian cancers [41]. As we have illustrated, cortactin was overexpressed in human lung cancer tissues compared with matched adjacent non-tumor tissues. Furthermore, the mean expression of cortactin was significantly lower in the group of patients with lymph node metastases compared with those without lymph node metastases. Functionally, decreased expression of cortactin was shown to decrease cell motility in a variety of assays, including transwell migration, wound closure, and cell motility. Moreover, our results indicated that cortactin expression was upregulated when lung cancer cells were treated with PDBu or HGF, as well as invadopodia formation in non-small cell lung cancer cells, which indicated an important role of cortactin in invadopodia formation. Furthermore, immunofluorescent staining showed that cortactin co-localized with F-actin in the newly-formed invadopodia of cells after PDBu or HGF stimulation. Besides, pharmaceuticals such as PP2, a Src inhibitor, were reported to inhibit phosphorylation of cortactin and inhibit the formation of invadopodia. Our findings indicated an important role of cortactin in invadopodia formation.

Cortactin has been reported to be regulated by several different microRNAs in different types of solid tumors. For example, Long et al. reported that miR-542-3p regulates cortactin in a targeted manner to modulate growth and invasion of colorectal cancer cells [42], while Zhang et al. reported that miR-182 inhibits proliferation and invasion of human lung adenocarcinoma cells via its effect on the human CTTN gene [31]. Additionally, Hong et al. reported that VEGF-C increased cortactin expression by down-regulating dicer-mediated maturation of miR-326, thereby relieving the suppressive effect of miR-326 on cortactin expression [43]. All these reports indicated that cortactin plays an important role on tumor invasion and is subject to regulation by microRNAs, which indicates that the mechanism of cortactin involved in cancer cell progression and invasion are complicated and obscure. Our findings revealed that miR-182 acts as a tumor suppressor molecule in metastasis and invadopodia formation by direct targeting of cortactin in NSCLC. The expression level of cortactin was inversely correlated with miR-182 expression in NSCLC samples. By targeting cortactin, miR-182 suppressed the Cdc42/N-WASP pathway as well as the Rac1 and Rock1 activities, and thus suppressed invadopodia formation.

MMPs are a large family of zinc-dependent endopeptidases that are capable of cleaving multiple ECM proteins. A lot of evidence suggests that MMPs play an important role in metastatic spread of tumor cells via ECM degradation [44]. MMP-2 and MMP-9, as key invadopodia proteases, have both been found to localize to invadopodia, where they mediate ECM degradation in vitro and in vivo [40]. It has been reported that cortactin plays multiple roles in delivering MMP2 and MMP9 proteases to degrade ECM in the cell invasion processes. In addition, cortactin was also reported to be an essential regulator of matrix metalloproteinase secretion and ECM degradation in invadopodia [6]. Our present study demonstrated that down-regulation of cortactin by miR-182 inhibited ECM degradation by invadopodia, manifested as down-regulating of ECM proteolysis, such as MT1-MMP, MMP2 and MMP9.

## Conclusions

Taken together, these findings reveal that targeting cortactin, and the subsequent suppression of invadopodia formation and ECM degradation, represents a novel mechanism for the action of miR-182. Thus, miR-182 might be a candidate for therapeutic application in NSCLC.

## Additional file

Additional file1: **Table S1.** Basic information of 55 patients with NSCLC. (PDF 64 kb)

## Abbreviations

CTTN: Cortactin
ECM: Extracellular matrix
HGF: Hepatocyte growth factor
MMP: Matrix metalloprotein
NSCLC: Non-small cell lung cancer
OS: Overall survival
PDBu: Phorbol 12,13-dibutyrate
PFS: Progression-free survival
PMSF: Phenylmethanesulfonyl fluoride
WASP: Wiskot-Aldrich syndrome protein
