# Supplementary material for: miR-182 suppresses invadopodia formation and metastasis in non-small cell lung cancer by targeting cortactin gene
Source: J Exp Clin Cancer Res. 2018 Jul 9;37:141. doi: 10.1186/s13046-018-0824-1 (PMC6038252; doi:10.1186/s13046-018-0824-1)
Supplement: Supplementary file 1 — Table S1. Basic information of 55 patients with NSCLC. (PDF 64 kb) [file 13046_2018_824_MOESM1_ESM.pdf]

Supplementary Table 1 Basic information of 55 patients with NSCLC

**Characteristic**		**N（%）**
Gender		
	Male	35 （63.6%）
	Female	20 （36.4%）
Age		
	>60	29 （52.7%）
	≤60	26 （47.3%）
Histology		
	AD	25 （45.5%）
	SCC	24 （43.6%）
	ASC	3 （5.45%）
	Others	3 (5.45%)
Smoking history		
	Nonsmoker	19 （34.5%）
	Smoker	36 （65.5%）
Metastasis		
	None	13 （23.6%）
	Yes	42 （76.4%）
Clinical Stage		
	I	2 （3.7%）
	II	32 （58.2%）
	III	13 （23.6%）
	IV	8 （14.5%）
